# Evaluating and Quantifying User and Carer Involvement in Mental Health Care Planning (EQUIP): Co-Development of a New Patient-Reported Outcome Measure

**DOI:** 10.1371/journal.pone.0149973

**Published:** 2016-03-10

**Authors:** Penny Bee, Chris Gibbons, Patrick Callaghan, Claire Fraser, Karina Lovell

**Affiliations:** 1 School of Nursing, Midwifery & Social Work, University of Manchester, Manchester, United Kingdom; 2 Cambridge Centre for Health Services Research, University of Cambridge, Cambridge, United Kingdom; 3 The Psychometrics Centre, Judge Business School, University of Cambridge, Cambridge, United Kingdom; 4 School of Health Sciences, Queen’s Medical Centre, University of Nottingham, Nottingham, United Kingdom; Yokohama City University, JAPAN

## Abstract

International and national health policy seeks to increase service user and carer involvement in mental health care planning, but suitable user-centred tools to assess the success of these initiatives are not yet available. The current study describes the development of a new reliable and valid, interval-scaled service-user and carer reported outcome measure for quantifying user/carer involvement in mental health care planning. Psychometric development reduced a 70-item item bank to a short form questionnaire using a combination of Classical Test, Mokken and Rasch Analyses. Test-retest reliability was calculated using t-tests of interval level scores between baseline and 2–4 week follow-up. Items were worded to be relevant to both service users and carers. Nine items were removed following cognitive debriefing with a service user and carer advisory group. An iterative process of item removal reduced the remaining 61 items to a final 14-item scale. The final scale has acceptable scalability (Ho = .69), reliability (alpha = .92), fit to the Rasch model (***χ***^2^(70) = 97.25, p = .02), and no differential item functioning or locally dependent items. Scores remained stable over the 4 week follow-up period, indicating good test-retest reliability. The ‘Evaluating the Quality of User and Carer Involvement in Care Planning (EQUIP)’ scale displays excellent psychometric properties and is capable of unidimensional linear measurement. The scale is short, user and carer-centred and will be of direct benefit to clinicians, services, auditors and researchers wishing to quantify levels of user and carer involvement in care planning.

## Introduction

Mental health services have undergone marked transformation over the last decade, reflecting a shift towards user-led models of care. Consolidation of contemporary care philosophies including patient-centred care [1], shared decision-making [2] and patient empowerment [3] have led to a rise in recovery-orientated services and a long standing drive towards increased service user and carer involvement in care planning. In the UK, this shift is most recently reflected in the personalisation agenda championed by adult social care, and gives rise to increasing demands for service implementation, local and regional evaluations and national-level audits focussed on service user and carer experience.

Independently, international health services and health service researchers have embraced a similar move towards increased development and use of patient-reported outcome measures (PROMs). Psychometrically robust PROMs are tools specifically designed to represent, prioritise and accurately quantify aspects of service provision from a service user perspective. Although not without criticism, data from PROMs are increasingly being incorporated into audits and research trials, and when aggregated and analysed at a systems level, provide a viable means by which to improve care quality and health outcomes across services and specialities [4,5].

Historically, local and national care quality surveys have monitored service user involvement in mental health care planning through objective performance indicators such as the presence of a service user’s signature on a care plan or the provision of a document copy for the person concerned [6]. Care plans often fail to include the views of service users or carers [7,8] and although contemporary policy [9] and guidelines [10] consistently advocate service user involvement as a means of improving the culture and responsiveness of mental health services,. evidence suggests that the majority of service users and carers continue to feel marginalised in the planning of their care [11,12].

Whether such data reflect genuine deficits in service user-involved care or merely discrepancies in the definition of this process is more difficult to elucidate. Despite its centrality to international and national mental health policy, user involvement in care planning remains inconsistently defined. Theory articulation allows for the conceptualisation of user involvement as both a linear (outcome-focused) and hierarchical (relational) event. Systematic evidence synthesis suggest that whilst current auditor standards emphasise and promote the documentary outcomes of mental health care planning, users and carers remain much more sensitive to the nature and quality of the relational aspects underpinning their development [12]. Thus, whilst existing measures do not prohibit meaningful user involvement, they may not fully reflect the practice-based activities that confer most benefit to service users and carers. Policy implementation and service improvement initiatives are thus potentially limited by a lack of validated and acceptable tools by which to quantify care planning involvement [13].

Published concepts of patient-centred care provide a potentially useful framework within which to locate a theoretical understanding of service user involvement in mental health care planning [14]. Systematic reviews of national and international mental health literature identify a common set of antecedents to care planning involvement [12]. These comprise the acquisition of adequate service user buy-in, meaningful information exchange, participatory deliberation and participatory decision-making. Existing frameworks of patient-centred care emphasise potential nomothetic (group level) and idiographic (individual level) influences on these processes, but are unable to fully capture the subtleties and nuances of frontline mental health practice.

Much of the literature that has been used to derive or validate theoretical frameworks of patient-centred care derives from physical healthcare contexts dealing with episodic rather than long term health conditions. Mental health services differ from physical health services in a number of discrete ways. Contemporary mental health services are borne of a unique service history founded on aspects of containment and compulsion. They are often required to work with a greater multiplicity of chronic and complex diagnoses, and can experience an entrenched stigmatisation of service users [15]. Thus, although there may be clear overlaps between user-involved mental health care planning and patient centred care, identical theoretical frameworks cannot be presumed.

A review of measures which assess patient perceptions of patient-centred care in family practice has heighted an issue with current measures insofar as they are often visit-based, thus limiting their relevance to the study of care processes over time [14]. This suggests that, even if the concepts of patient-centred care *were* comparable in both mental and physical illnesses, present measures may serve limited purpose for service improvement and research.

A recent systematic review of patient reported measures of service user involvement [13] has revealed a lack of care-planning measures that are able to meet service user nominated acceptability criteria alongside published (EMPRO) standards for psychometric quality. The increasing use of PROM data to assist with evidence based healthcare decisions and commissioning has led to a greater mandate for these PROMs to function as accurate and reliable measurement tools. The majority of PROMs have been developed using classical test theories (CTT), which are widely regarded as out-dated and which can only yield ordinal-level scores from questionnaires [16,17]. Reliance on ordinal measures may lead to inconsistencies and inaccuracies in decisions supported by these PROMs [18,19]. Modern test theories (MTT), including item response theory, the Rasch model and Mokken analysis, offer the statistical complexity to create interval-level measures and increase confidence in decisions made using measures derived under these paradigms [17].

The objective of this study was to i) co-develop, with users and carers, a new PROM to assess user/carer involvement in mental health care planning and ii) investigate its psychometric and scaling properties via a combination of Classical Test, Mokken and Rasch analyses.

## Methods

All potential participants were provided with a participant information sheet approved by service users and written to current UK National Research Ethics Service (NRES) guidelines. This information sheet provided participants with information about the study, including the potential benefits and risks of taking part. Contact details were provided for the research team and all participants had the opportunity to speak to a researcher before deciding whether or not to take part. Due to the potentially sensitive nature of the topic area (i.e. personal appraisal of health services received), participant anonymity was preserved. Instead informed consent was implied by participants choosing to return their questionnaires to the research team. This study, and the informed consent procedure, were approved by the UK NRES Committee (East Midlands: Nottingham 2) in January 2014 (Ref: 13/EM/0364).

### Measure design and item development

Candidate items were developed from 74 interviews and 9 focus groups conducted with service users, carers and mental health professionals recruited from two large National Health Service (NHS) Trusts in North West and Central England. In the UK, NHS Trusts are statutory service providers to which patients are assigned based on geography.

Seventy candidate items were developed. Face validity was examined with a mixed sample of 16 members of a service user and carer advisory group (SUCAG) using cognitive interviewing [20]. Nine items were removed because the SUCAG found their language or wording unclear or hard to understand. The remaining 61 items comprised the nascent scale. Members of the SUCAG were also asked to comment on potential response formats. Consensus was reached for a 5-point Likert scale with named anchors of ‘Strongly disagree’ and ‘Strongly agree’ and a middle neutral value with the label “Neither agree nor disagree.”

### Questionnaire administration

Service users were defined as individuals diagnosed with a severe and enduring mental health condition e.g. schizophrenia, other psychotic illnesses, borderline personality disorder and other personality disorders or severe affective disorders including severe unipolar depressive disorder, and who were in contact with statutory (NHS) secondary care mental health services. In the UK, secondary care mental health services for severe and enduring mental illness are typically comprised of inpatient and community mental health care. Carers were individuals who self-identified as caring for a service user with a severe and enduring mental health condition as defined above.

Recruitment strategies included advertising on NHS Trust intranets, newsletters and press releases, posters displayed within Trust premises, local Trust-based and third sector study advocates and local/national user/carer forums. Service user/carer participants from earlier interviews and focus groups were also invited to take part. Data were collected using online, postal and face-to-face modalities. Postal and face-to-face completion was undertaken across five NHS trusts in North-West and Central England. Support for data collection was provided by the members of the research team and the UK Mental Health Research Network. An online version of the nascent PROM was developed using subscription-based online questionnaire hosting service SelectSurvey and promoted on the University of Manchester’s School of Nursing, Midwifery and Social Work website. Invitations to complete the PROM were sent out via Twitter and ‘re-tweeted’ by local and national mental health charities in the UK.

Respondents were invited to complete the PROM if they, or a person they cared for, had ever received a care plan. The questionnaire was preceded with introductory text designed to orientate participants to its purpose. The term care plan' referred to the service user’s main care plan, or in the event of multiple documents, their most recent care plan.

### Test-Retest & scale validation

A prior systematic review of user and carer involvement measures was unable to identify any questionnaires that were of high quality or acceptable to users and which could be used as a comparator for construct validity [13]. In this study, the validity of the ‘involvement in care planning’ construct was ensured by extensive collaboration with service users, carers and professionals at multiple stages in the development of the initial item bank and strict tests of unidimensionality in the final Rasch analysis [21]. A randomly-selected sample of 40% of those who had completed the scale were approached to complete the 61-item measure approximately four weeks after baseline to assess test-retest reliability.

### Sample size

For Rasch analysis, a minimum sample size of 250 allows for over 99% confidence that item calibrations are stable to within ±0.5 logits, irrespective of scale targeting [22,23]. This minimum sample size was also deemed sufficient for the other planned psychometric analyses detailed below.

### Data cleaning

Prior to statistical analysis data were double-entered and a 5% accuracy check was made. Fewer than 0.1% errors were detected during the double entry procedure. Thirteen records (3.13% of the total sample) were removed from the dataset for having >40% missing PROM answers (≥24/61 items), leaving 402 for analysis. The paper-based questionnaire was arranged to have approximately 40% of the items on the first two pages, so this criterion was decided upon to eliminate page turning error. Eight of the removed records did not complete any of the PROM items (but did complete the demographics) and the remaining five completed the first page, but not other items. The mean number of missing items was 0.87 (SD = 2.68) for the remaining records in the dataset

### Psychometric and statistical analyses

#### Exploratory factor analysis

An exploratory factor analysis based on a polychoric correlation matrix was conducted to establish initial dimensionality. The factors were rotated using oblique Promax rotation. Significance of factors was determined using the Very Simple Structure procedure [24]. Very simple structure was chosen as it is a parsimonious technique for factor determination, which could be used to establish initial dimensionality prior to more rigorous and accurate tests of dimensionality in both Mokken and Rasch analyses. Mokken analysis is better suited to analysing unidimensional scales with bi-polar conceptual structure than factor analysis [25,26]. This approach was taken in part to avoid the creation of illusionary factors caused by items clustering at different performance levels as different factors [27]. Analyses were conducted with the ‘psych’ [28] and ‘polycor’[29] packages for the R statistical computing environment [27].

#### Mokken analysis

Mokken analysis [30], a non-parametric approach, can identify if the structure of the items will be consistent with the Rasch model. A Loevinger’s coefficient value of < .30 would indicate an item that was inconsistent with the structure of the scale [31]. The Mokken Automated Item Selection Procedure (AISP) was also used to explore the presence of an additional factor, in addition to those revealed using EFA.

#### Rasch analysis

Rasch analysis [32] was conducted in order to derive a final linear unidimensional measure of care planning involvement. Whilst a number of Rasch-like item response theory approaches exist (e.g. Samejima’s Graded Response Model), the Rasch model’s proven ability to develop and validate questionnaires that satisfy the demands of fundamental measurement and are capable of creating interval-level measurement [33] made it perfectly suited to the current study. In addition, measures developed using the Rasch model tend to be brief [34,35] which was an important concern raised during the service user and carer interviews that preceded this study. Interval-level measurement is a necessity if accurate comparisons are to be made between patients or across patients over time, or if mathematical operations are to be carried out with questionnaire data [36,37].

Rasch analysis provides additional tests alongside traditional assessments of validity and reliability, including local independence of items, differential item functioning (DIF), item category threshold order, unidimensionality and scale targeting [21,38,39]. Scale improvement was carried out using an iterative process of item removal. The iterative process involved assessments of category threshold order, item fit to the Rasch model (chi square p>0.01), assessment of fit residuals (fit residuals within ±2.5), local dependency (residual correlations < .10) and differential item functioning (no significant ANOVA interactions by demographic group). Items that violated any of the above assumptions were individually removed and the remaining items were re-analysed. This process was repeated until good scale fit to the Rasch model was achieved and no items presented category disordering, misfit to the Rasch model, high fit residuals, local dependency or differential item functioning. Further details for each of these diagnostic tests are presented below.

#### Category threshold ordering

When the assigned category order (Likert scale) does not accord with the latent variable (care planning involvement) then the empirical measures for each category are out of sequence and there is misfit [40,41]. Where category thresholds were disordered (indicated by visual analysis of item threshold curve graphs) adjacent categories were ‘collapsed’ into a category. For example if an item scored 0-1-2-3-4 had disordered thresholds between response categories 2 and 3; it would be rescored 0-1-2-2-3.

#### Item fit to the Rasch model and fit residual

Estimates of a location on a common metric are provided for both persons (ability) and items (difficulty). In the context of the current study, ‘ability’ may be understood to represent the amount of care planning involvement the respondent has experienced and ‘difficulty’ may be understood to represent the level of care planning involvement represented by the item. When data are analysed using the Rasch model, both the items and persons are calibrated and presented on the same metric which is measures in logits. This allows the location of people and items to be compared, and allow judgements to be made about scale targeting. Scale targeting is considered to be acceptable if the majority (90%) of people responding to the scale fall within the measureable range of the scale [42]. Occasionally floor and ceiling effects may be tolerated if a scale provides good measurement around important clinical cut-off points [43].

#### Local dependency

One of the key assumptions of psychometric theory is that the relationship between items is solely attributable to the specific latent trait. However items in nascent scales commonly continue to be related to one another, after accounting for their individual contribution to the latent trait. The effect of local dependency on the ability estimates within a scale are substantial and therefore a strict criterion of positive residual correlation between the items >.10 is used [34,44].

#### Differential item functioning

Differential item functioning occurs where different demographic groups within the same sample respond in a systematically different way to a certain item irrespective of their location on the underlying trait [39]. In the current study DIF was assessed for relationship to services (*i*.*e*. service user/carer/both), age (19 to 36,37 to 47,48 to 58, 57 plus) and gender (male/female). Differential item functioning is detected using analysis of variance (ANOVA, 5% alpha) with bonferroni correction for multiple comparisons.

#### Scale Reliability

Reliability is assessed using the Person Separation Index (PSI), which reflects the extent to which items can distinguish between distinct levels of functioning (where 0.7 is considered a minimal value for research use; 0.85 for clinical use)[45]. Where the distribution is normal, the PSI is equivalent to Cronbach’s alpha.

#### Unidimensionality

Independent t-tests are employed to confirm the assumption of unidimensionality for the final scale. Principal component analysis of the residuals is conducted to identify two subsets. These subsets are then compared and the number of significant t-tests outside the ± 1.96 range indicates whether the scale is unidimensional or not. Unidimensionality is indicated when fewer than 5% of the t-tests are significant (or the lower bound of the binomial confidence interval overlaps 5%) [21,38].

Rasch analyses were conducted using the partial credit polytomous model with conditional pair-wise parameter estimation [35] as response categories were polytomous (*i*.*e*. >2 response options). Analyses were conducted using SPSS 22 [46], the ‘mokken’ package [47,48] for the R Statistical computing environment [49] and RUMM2030 [50].

#### Test-retest reliability

Test-retest reliability was assessed using correlations between interval-level scores for the 14-item measure at baseline and four week follow-up for each of the three subscales.

## Results

Sample demographics are presented in the S1 Table

### Exploratory factor analysis

Very simple structure (VSS) analysis revealed a single interpretable factor. The VSS was maximised with a single factor (VSS = .97). Forty six items loaded onto the first factor, with 15 items loading below .30 or onto other factors.

### Mokken analysis

The scalability of the 46 remaining items scale was assessed using Mokken analysis. Good scalability was confirmed for all but one item (Item 57: Care plan decisions seem to be made by just one person). This item was removed as it returned a Ho value of .22, below the .30 criterion. Mokken’s AISP procedure did not identify any additional factors for the remaining 45 items.

### Rasch analysis

Following exploration of dimensionality and scalability, the 45 remaining items were analysed using Rasch analysis. The 45 items did not fit the Rasch model (*x*^2^ = 6622.15, p < .001), leading to an iterative process of item modification and deletion. Likelihood-ratio tests confirmed the suitability of the unrestricted partial credit Rasch model for this dataset.

### Category threshold analysis

**Fig 1:**
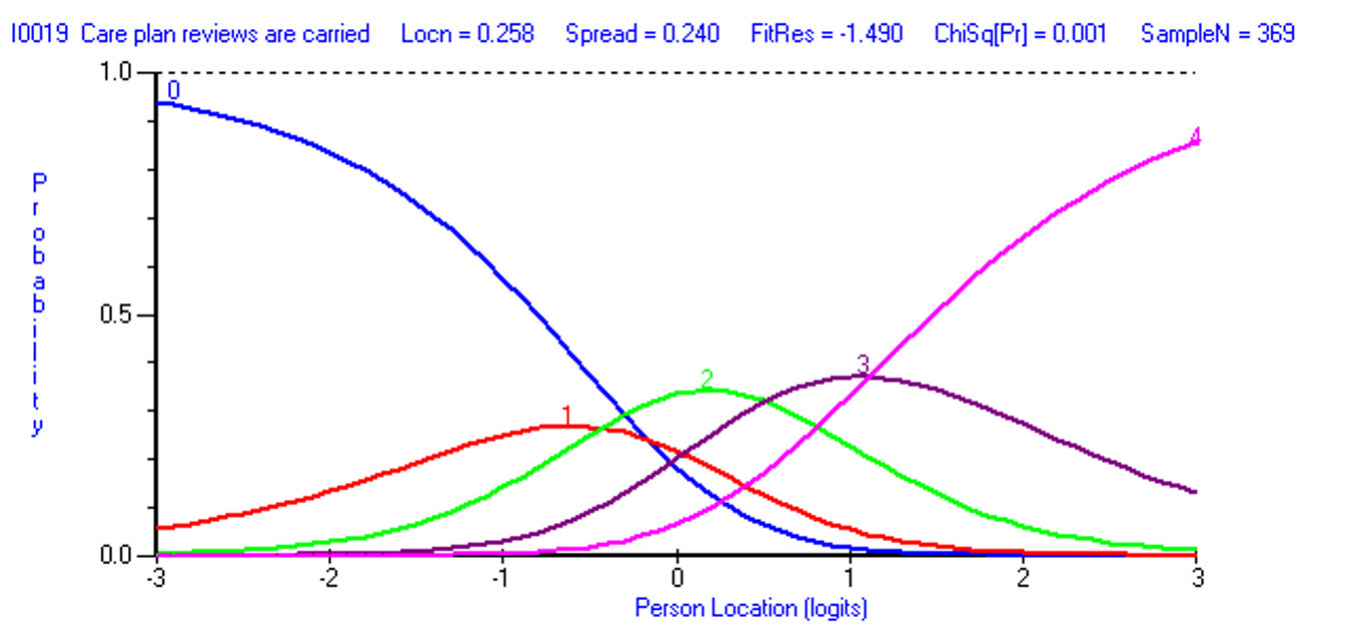
Disordered category thresholds for Item 19 (Care plan reviews are carried out in good time).

The suitability of the 5-point Likert scale was assessed for all items. In total 31 items had disordered thresholds, indicating that respondents were unable to distinguish between two or more response options for each question. For the majority of the misfitting items on the scale, respondents did not discriminate between response categories 0 (Strongly disagree) and 1 (Disagree; see Fig 1). Participants also had difficulty discriminating between categories 2–3 (3 items) and 3–4 (3 items). Items were re-scored appropriately before continuing with the analysis.

### Misfitting items

**Fig 2:**
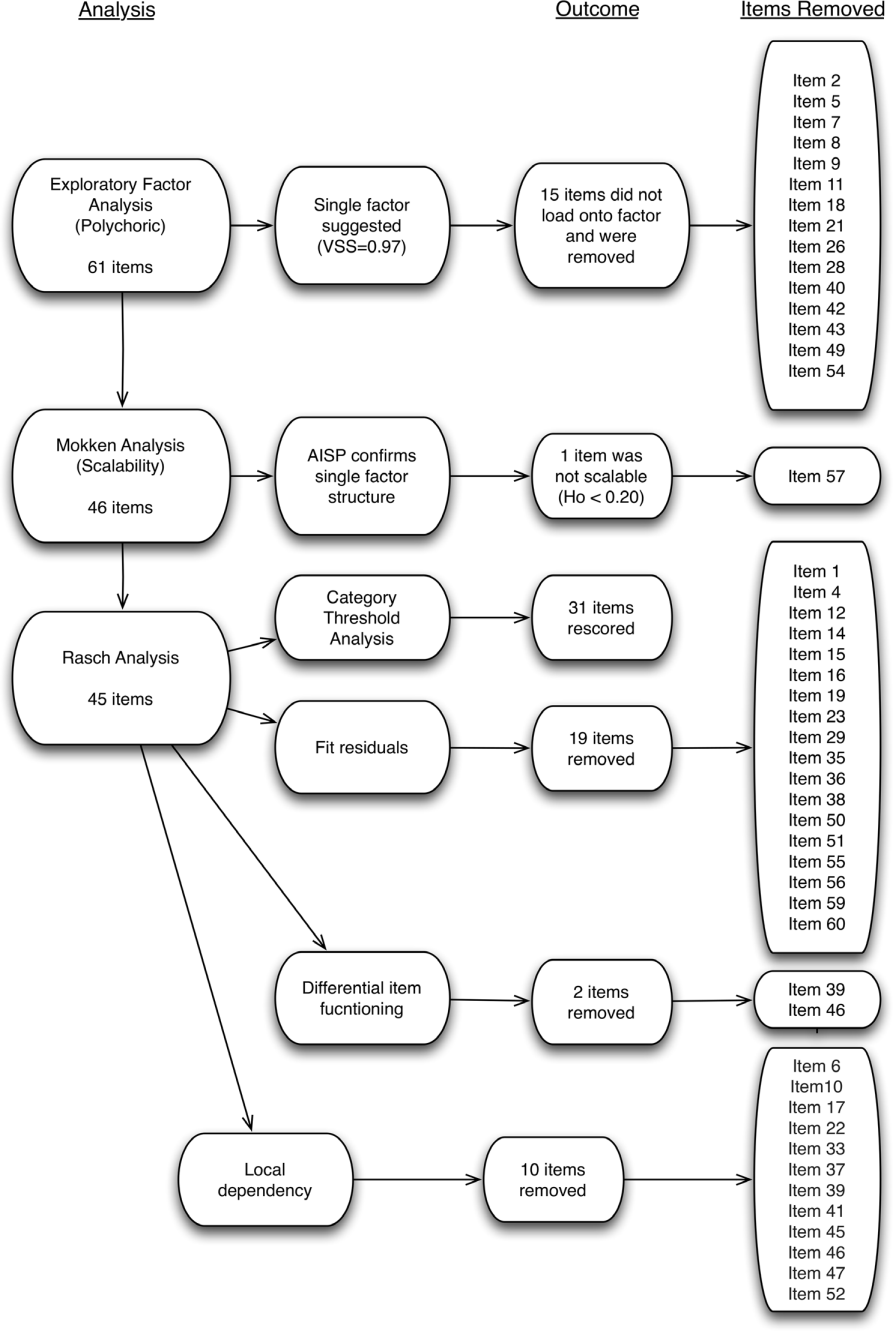
EQUIP Analysis Overview.

Following re-scoring items with disordered thresholds, individual item fit statistics were analysed. A number of items displayed misfit to the Rasch model or fit residuals beyond the ±1.4 range. An iterative process of item removal led to the 19 items being removed from the scale. The items which were removed from the scale, and the reasons for their removal, are detailed in Fig 2.

### Differential item functioning

**Fig 3:**
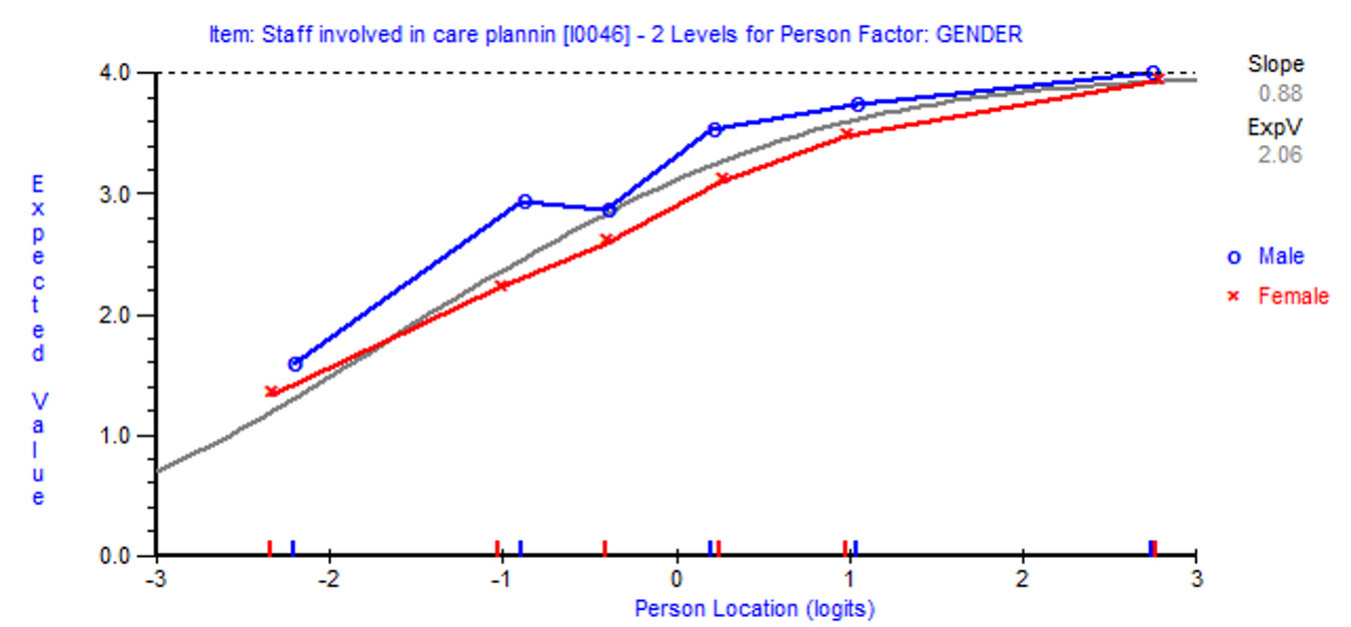
Differential item functioning by gender for Item 46 (Staff are helpful, kind and polite).

Two items displayed differential item functioning. Item 39 (The care plan is unique to me) displayed non-uniform DIF by different age groups (*f*(3), 7.03 p<0.001). For Item 46 (Staff involved in care planning are helpful, kind and polite), men scored uniformly higher than women at every level of care planning involvement (*f*(1), 14.02, p<0.001; see Fig 3). No item displayed DIF between service users and carers.

### Local dependency

Ten items displayed local dependency, leading to an iterative process of removing items that had multiple dependencies.

### Final scale

**Fig 4:**
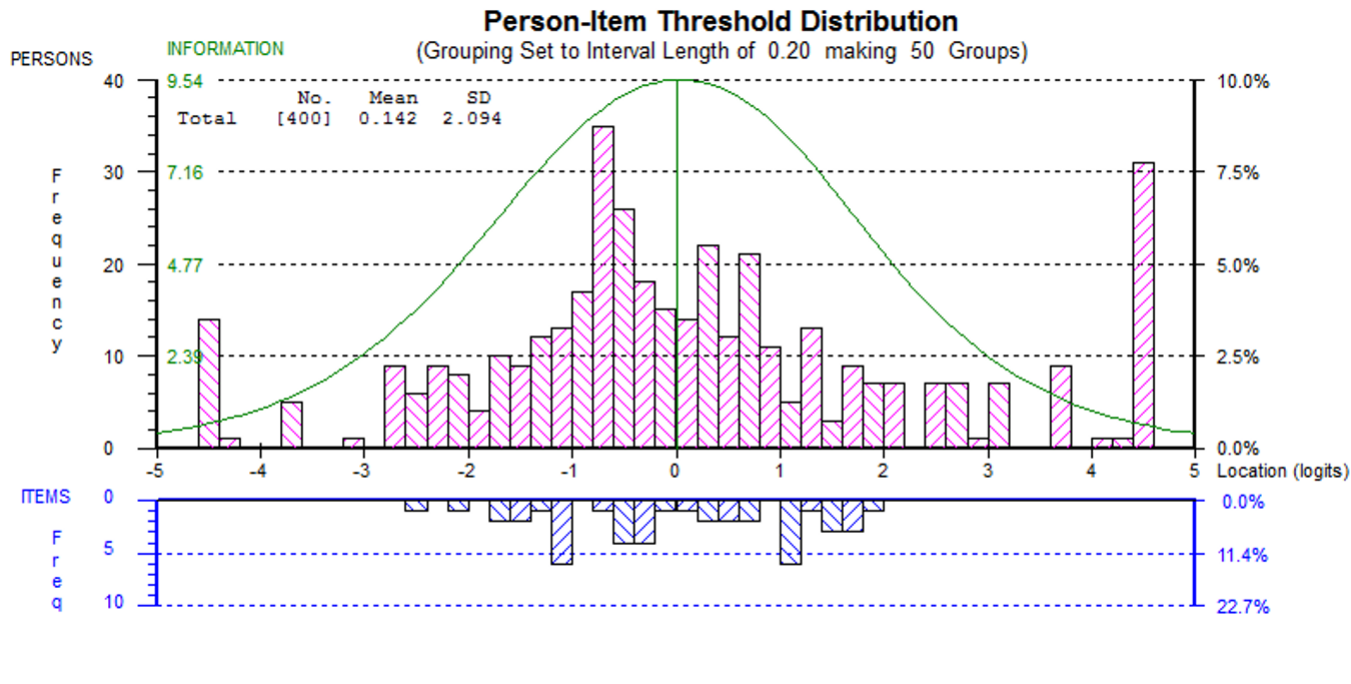
Person and Item Distribution for the Final EQUIP measure.

The final 14-item scale showed acceptable fit to the Rasch model (*x*^2^ = 97.25, p = 0.02) including excellent person-separation reliability (PSI = .93; see S2 Table). Mean person and item fit residuals were acceptable and the 14-item scale had good dimensionality (3.76% of t-tests significant). Scale targeting was 11.94%, slightly beyond the ideal value of 10%. Fig 4 shows the distribution of persons and items. The majority of the extreme scores were at the ceiling of the scale (indicating excellent involvement in care planning). Using Mokken analysis, the final scale also returned a Loevinger’s scalability coefficient of 0.70 (SE = .018). Fig 3 presents an overview of the analysis, and the final items retained. The items that comprise the final scale alongside un-centralised threshold values are shown in the S3 Table. The final scale is presented in Table 1.

**Table 1:** The final 14-tem EQUIP Scale.

Analysis number	New number	Wording	Scoring				
3	1	The care plan has a clear objective	0	0	1	2	3
13	2	I am satisfied with the care plan	0	0	1	2	3
20	3	I am happy with all of the information on the care plan	0	0	1	2	3
22	4	The contents of the care plan were agreed on	0	0	1	2	3
25	5	Care is received as it is described in the care plan	0	0	1	2	3
27	6	The care plan is helpful	0	0	1	2	3
30	7	My preferences for care are included in the care plan	0	1	2	3	4
31	8	The care plan is personalised	0	0	1	2	3
34	9	The care plan addresses important issues	0	0	1	2	3
44	10	The care plan helps me to manage risk	0	0	1	2	3
48	11	The information provided in the care plan is complete	0	1	2	3	4
53	12	The care plan is worded in a respectful way	0	0	1	2	3
58	13	Important decisions are explained to me	0	0	1	2	3
61	14	The care plan caters for all the important aspects of my life	0	0	1	2	3

### Test-rest reliability

The test-restest questionnaire was completed by 57 patients. High Pearson’s correlation values between scores provided evidence of invariance over time (r_*p*_ = .88, p<0.001).

### Raw score to interval-scale conversion

The table in S4 Table provides a nomogram which may be used to convert ordinal scores gained from the EQUIP questionnaire into interval-level trait estimates, provided that data are normally distributed and complete.

## Discussion

Promoting shared decision making and involving service users and carers in care planning are central to national policy initiatives aimed at optimising recovery and improving the quality of mental health care [12]. Yet, despite long standing support for the ideology of user/carer involvement, patient-reported measures of participatory care are lacking. Current measures, such as those used by the UK Care Quality Commission (CQC), focus on objective indicators of care planning administration rather than those aspects of care planning that service users value most. A previous systematic review of user- and carer involvement in mental health care-planning has demonstrated a lack of high quality outcome measures that are grounded in service user values and acceptable to users/carers in terms of completion [13]. The current study aimed to address this methodological and translational gap.

Care planning inevitably necessitates interactions between different stakeholder groups and the context and quality of these interactions will impact directly on the way in which the meaning of the event is construed. The EQUIP PROM was developed in collaboration with service users and carers and conforms to the highest standard of evaluative analysis. The final measure is 14-items long and provides a unidimensional measure of service user and carer involvement in mental health care planning. The 14-item scale has exceptional psychometric attributes, satisfying the strict demands of the Rasch model and displaying excellent reliability. Quantifiable performance indicators are advantageous to quality improvement and clinical research and in its current form the scale will add considerable value to trials, service evaluations and audit. Future development of the EQUIP PROM will focus on development of computer adaptive testing, which may in turn facilitate quicker questionnaire completion times and provide instant graphical feedback to health professionals and users.

The stringent, methodological process that was followed in our study led to an initial measure of 61 items, that were originally developed in conjunction with service users and carers, being reduced to a 14-item psychometrically validated PROM. Measure length and ease of completion is identified as a key user-nominated attribute for PROM acceptability in this population. Nonetheless, the utility of any measure depends on its validity, reliability, sensitivity and feasibility of completion, and a trade-off between these criteria is often necessary [1]. It is possible that some concepts that were originally conceived as important to service users during item generation were not adequately represented by the items retained in the final measure. This accepted, the final measure encompassed a breadth of items that represented a multiplicity of user responses. Key aspects of patient-centred care identified as theoretically important by other studies were represented. These included holistic approaches to care (items 14 and 11); the quality of the user-professional relationship (item 12) and personalisation and relevance to user experience (items 6, 8 and 9). Specific antecedents to care planning involvement were also included in relation to information exchange (item 3), participatory deliberation (item 7,13) and shared decision making (item 4). Additional items sought to quantify satisfaction with specific and unique outcomes of the care planning process, including written documentation (i.e. the care plan) (items 1 and 2), and the mental health care and longer term self-management skills that result (items 5 and 10).

The emergence of differential item functioning for two items in the original item bank is an interesting finding that demands further exploration. Whilst item 39 (The care plan is unique to me) functioned differently by different age groups, item 46 (Staff involved in care planning are helpful, kind and polite), demonstrated significantly lower levels of agreement among females than males. The true relationships between user characteristics and their expectations and/or experiences of care planning are difficult to establish. The potential for these data to reflect genuine rather than perceptual differences in care planning participation is supported by small scale studies that suggest service users’ and carers knowledge may be mediated by demographic or ethnic status [11]. Future analyses are needed to model potential relationships between the EQUIP PROM scale and external measures, including demographic, clinical and psychological variables. The critical test lies in ascertaining the validity of the scale in predicting other pertinent outcomes such as overall health service quality, service satisfaction and health care utilisation. Routinely collecting data and understanding such relationships are likely to be key to developing, implementing and evaluating new initiatives to enhance user/carer involvement in mental health care planning.

## Supporting Information

S1 Table Sample demographics. (XLSX)

S2 Table Rasch fit statistics. (XLSX)

S3 Table Rasch delta statistics. (XLSX)

S4 Table Nomogram. (XLSX)
